# Does the Use of Oral Nutritional Supplements Influence the Rate of Postoperative Complications After Uniportal Video-Assisted Thoracoscopic Lung Resection?

**DOI:** 10.3390/jcm14124226

**Published:** 2025-06-13

**Authors:** Marco Andolfi, Michela Tiberi, Michele Salati, Marina Taus, Nadia Campelli, Francesco Xiumè, Alberto Roncon, Gian Marco Guiducci, Anna Chiara Nanto, Claudia Cola, Loris Angeli Temperoni, Majed Refai

**Affiliations:** 1 Thoracic Surgery Unit, AOU of Marche, 60126 Ancona, Italy; 2 Dietetic and Clinical Nutrition Unit, AOU of Marche, 60126 Ancona, Italy

**Keywords:** uniportal video-assisted thoracoscopic surgery (uVATS), oral nutritional supplement, minimally invasive surgery, postoperative complications, ERAS

## Abstract

**Background:** The positive effects of oral nutritional supplements (ONS) on postoperative outcomes have been well recognized in several previous studies. However, to date, little data has been available with respect to determining the best immune modulating supplement to use and what its impact might be in thoracic surgery. The aim of this study was to evaluate the role of preoperative immune-nutritional supplement intake as predictor of postoperative cardiopulmonary complications (CPCs) in patients undergoing uniportal video-assisted thoracoscopic (uVATS) lung resection. **Methods:** This is a retrospective, observational study enrolling consecutive patients who underwent uVATS lung resections for cancer from January 2022 to December 2024 in the context of the Enhanced Pathway of Care (EPC) Program. All patients were evaluated by a nutritionist and dietetics team during the preoperative phase. The nutritional protocol consisted of 250 mL ONS rich in arginine, omega-3-fatty acids, and nucleotides to be taken twice a day for 5–7 days before surgery. **Results:** Four hundred ninety-one patients were enrolled: 277 patients underwent anatomic lung resection and 214 underwent wedge resection (WR). Utilizing the univariate analysis, we found that in patients undergoing anatomic lung resection, not-ONS-intake, high Body Mass Index (BMI), and arrythmia were correlated with a higher CPCs rate compared to the patients without nutritional supplementation (7.2% ONS vs. 15% not-ONS, *p* = 0.04; BMI 28.4 kg/m^2^ vs. BMI 26.4 kg/m^2^, *p* = 0.03; 31.2% arrythmia vs. 9.4% no-arrythmia, *p* < 0.01). These correlations, except for BMI, were confirmed after stepwise logistic regression. Alternately, in patients undergoing WR, hypertension and low-FEV1% were associated with a higher CPCs rate (5.1% hypertension vs. 0.4% no-hypertension, *p* = 0.02; FEV1% 79.7% vs. 95%, *p* = 0.01). **Conclusions:** Our results demonstrated that preoperative ONS after uVATS anatomic lung resection, in the context of an EPC program, influences the postoperative period, reducing the CPCs rate.

## 1. Introduction

Many studies have reported a close association between nutritional status and postoperative outcomes in patients undergoing lung resection for cancer [1,2,3], demonstrating that the poor immune–nutritional status was a predictor of complications [4].

Indeed, cancer surgery increases patients’ catabolism and energy needs, in addition to inducing excessive production of inflammatory cytokines with consequentially severe alterations to the immune response system [5]. Indeed, major surgery has been shown to eventuate in an increase in prostaglandin E_2_-synthesizing monocytes and a simultaneous decrease in functionally competent CD3- and CD4-positive lymphocytes, producing a shift in the Th1/Th2 balance toward a Th2 response and thus interfering in the cell-mediated and humoral immune systems [6].

Moreover, some authors have demonstrated that the decreased expression of human leukocyte antigen (HLA)-DR on monocytes caused by the surgical trauma may lead to a temporary immunodepression, which can be responsible for an increased rate of postoperative complications and an increase in mortality [7,8].

Based on these findings, most studies have shown that nutritional support treatments prior to oncological surgery can reduce inflammatory markers and, consequently, postoperative adverse events [9]. Therefore, The European Society for Parenteral and Enteral Nutrition (ESPEN) and the American Society for Parenteral and Enteral Nutrition (ASPEN) recommend immunomodulating nutrition administration in the preoperative period in cancer patients in order to better clinical outcomes [10,11]. However, to date, little information focusing on the indication, the timing, and the duration of preoperative treatment in thoracic surgery is available [12].

In the present observational study, we aimed to investigate, within the context of an enhanced pathway of care (EPC) program, the potential predictors of postoperative complications in patients undergoing uniportal video-assisted thoracoscopic surgery (uVATS) lung resection for non-small cell lung cancer (NSCLC) and metastatic disease. Specifically, we verified whether the use of preoperative oral immune–nutritional supplementation was associated with a lower cardiopulmonary complications (CPCs) rate.

## 2. Materials and Methods

### 2.1. Study Design and Patient Information

This retrospective, observational study involving human participants was presented to the local ethical committee for notification even though, due to the retrospective nature of the work, formal approval was not required.

In the present study we enrolled 491 consecutive patients affected by neoplastic disease undergoing uVATS lung resection at our institution from January 2022 to December 2024.

All enrolled patients were informed by medical staff and signed informed written consent to the use of their anonymized data for scientific purposes.

The baseline characteristics and medical history of each patient were assessed at the preoperative visit 15 to 20 days before the operation.

The indication at the surgical treatment was defined by a dedicated multidisciplinary certified meeting with thoracic surgeons, oncologists, pneumologists, radiologists, and pathologists as participants.

The functional assessment performed to determine the suitability of surgical treatment followed all relevant internationally published protocols [13].

The patients’ operative and postoperative management was standardized using the institutional EPC, as elsewhere described [14,15].

Patients were considered eligible for inclusion in the present study if they were ≥18 years of age, their American Society of Anesthesiologists (ASA) score was ≤3, and they were scheduled for elective uVATS lung resection for lung cancer or metastatic disease. Patients were excluded from the analysis if their ASA score was >3; there was a conversion to open surgery; there was planned postoperative intensive-care monitoring; there was no caregiver, there was an inability to collaborate (language barriers, psychiatric pathology), or they were malnourished (Body Mass Index, BMI <18.5 kg/m^2^), in order to form a homogenous group and ensure that the patients’ nutritional levels were not affected by any factor other than the operation.

During the preoperative phase, all patients received a nutritional evaluation by a specialized team. In particular, patients were informed regarding their immune–nutritional status (based on the clinical and laboratory assessment) and, if not presenting chronic renal failure, obesity, uncontrolled diabetes, overweight status, or weight gain in the last months, received 250 mL oral nutritional supplementation (ONS) containing 9.6 g of arginine, 1.76 g of omega-3 fatty acids, and 0.96 g of dietary nucleotides to be taken twice a day for 5–7 days before surgery.

All patients were given an instruction booklet with instructions about the EPC protocol. The same booklet was also used to track preoperative compliance with the dietetic protocol as well as potential side effects.

### 2.2. Outcomes

In order to evaluate the role of ONS use as a predictor of complications after uVATS lung resection, we assessed its association with the postoperative CPCs rate. We considered the following CPCs, with standardized definitions as reported in the literature [16]: pneumonia, atelectasis requiring bronchoscopy, respiratory failure, reintubation, acute respiratory distress syndrome, pulmonary embolism, pulmonary edema, acute myocardial ischemia, arrhythmia, acute cardiac failure, stroke or transient ischemic attack, and acute kidney disease. We assessed the complications that arose within the first 30 days after the operation and/or during the entire hospital stay.

### 2.3. Surgical Procedure

The scheduled procedures were all performed by staff surgeons. As previously reported [14], surgical procedures were performed in a lateral position, through a single 3–4 cm incision performed at the 5th intercostal space, anterior axillary line.

A wound protector was applied, and a camera (5 mm, 30°) was introduced in the same incision with no trocar. Thoracoscopic instruments were routinely used. In all cases, at the end of the procedure, only one 24 Ch chest tube was placed in the posterior part of the incision and connected with a digital drainage system. Intraoperative pain was controlled by infiltrating three intercostal spaces (IV, V, and VI intercostal spaces) with Naropine 7.5% at the end of the operation under thoracoscopic vision.

### 2.4. Statistical Analysis

Data for the current analysis was sourced from our institution’s prospectively maintained database. Prior to analysis, we performed a tailored data quality assessment and cleaning procedures. We decided to exclude from the analysis the variables with a completeness rate below 90% as well as subjects having missing data on the primary outcome (cardiopulmonary complications). On the other hand, specific imputation techniques were implemented for variables with an incompleteness rate of up to 10%.

Normality tests for numeric variables were conducted using the Shapiro–Wilk test. The Student *t*-test was applied to numeric variables with normal distribution, and the Mann–Whitney test was used for those without normal distribution. The Chi-square test assessed categorical variables, provided no cell contained fewer than 10 observations.

Univariate analysis investigated the potential association between preoperative and operative factors and postoperative CPCs.

A stepwise logistic regression including parameters with *p* ≤ 0.1 in the univariate analysis was subsequently performed to identify independent predictors of complications.

## 3. Results

All 491 patients were treated with the uVATS approach (48% male, mean age 67.6 years old). Among these, 277 patients underwent anatomical lung resection (232 lobectomies, 41 segmentectomies, and 4 bilobectomies), while 214 underwent wedge resection (WR). In the final pathological findings, all patients presented a neoplastic disease: 383 NSCLC, and 108 with metastatic disease. Among these 491 patients, 45 (9.2%) patients developed CPCs. Thirty-four patients presented pulmonary complications (eight had acute respiratory failure; one, pneumonia; and twenty-five, prolonged air leaks). Eleven patients had cardiovascular complications (five, arrhythmia; two, pulmonary embolism; one, syncope; one, hemothorax; one, cerebri ictus; and one, hypertensive peak). In the univariate analysis, in evaluating potential association between preoperative/operative factors and postoperative CPCs, we found that not-ONS-intake, high BMI, and arrythmia in patients undergoing uVATS anatomical lung resection were correlated with a higher CPCs rate compared to the control group (ONS, *p* = 0.04; BMI, *p* = 0.03; arrythmia, *p* < 0.01; see Table 1). Specifically, thirty-three of the two hundred seventy-seven patients (11.9%) presented a postoperative adverse event, and among these, eight received ONS (7.2% vs. 15% not-ONS patients) and ten had preoperative arrythmia (31.2% vs. 9.4% not-arrythmia). The BMI mean value in these 33 complicated patients was 28.4 kg/m^2^, while in non-complicated ones it was 26.4 kg/m^2^.

These correlations, except for BMI, were confirmed after stepwise logistic regression (dependent variable: CPC; independent variables: age, BMI, arrhythmia, diabetes, ONS), demonstrating that ONS assumption and arrythmia were independent predictors of complications in patients undergoing uVATS anatomic lung resection for cancer (see Table 2).

On the other hand, in patients undergoing WR, hypertension and low FEV1% were associated with a higher CPCs rate (5.1% in hypertension pts vs. 0.4% in non-hypertension pts, *p* = 0.02; FEV1% 79.7% vs. 95%, *p* = 0.01; see Table 3). In this subgroup of patients, only twelve of two hundred fourteen patients (5.6%) recorded a CPC and, among these, six patients received a preoperative ONS (6.4% vs. 4.9% not-ONS patients), that resulted not associated with a low rate of postoperative adverse events (*p* = 0.64). However, due to the very limited number of observed events, this determination cannot fully elucidate the independent-predictor role of this finding without incurring the risk of underestimating its clinical impact.

Considering the role of ONS as strong predictor of CPCs after anatomic lung resection, we verified the baseline characteristics of the two groups of patients (ONS vs. not-ONS), consisting of 204 and 287 patients, respectively, and we found that they were homogeneous in terms of sex, FEV1%, ASA score, cardiovascular and cerebrovascular disease, diabetes, chronic kidney and liver disease, and type of resection. However, they showed statistically significant differences regarding age (69 years old in ONS group vs. 67.6 years old in not-ONS group, *p* = 0.01), BMI (24.2 kg/m^2^ in ONS group vs. 27.9 kg/m^2^ in not-ONS group, *p* < 0.01) and DLCO% (73.1% in ONS group vs. 76.9% in not-ONS group, *p* = 0.01). Table 4 summarizes the baseline characteristics of the enrolled patients.

As reported in the Table 4, 287 patients did not receive ONS. The reasons were obesity (one hundred thirty-one patients, 46%), overweight status (one hundred two patients, 36%), recent weight gain (twenty-four patients, 8%), chronic renal failure (eight patients, 3%), uncontrolled diabetes (ten patients, 3%), the patient’s decision (six patients, 2%), and side effects (six patients, 2%: two for early diarrhea onset and four for mild or severe nausea). Otherwise, all 204 patients who received ONS observed the prescribed dose of 500 mL (2 × 250 mL) per day for 5–7 days before surgery.

Five patients required a reoperation: four for aerostasis and one for haemostasis. Neither in-hospital nor 30/90-day mortality was recorded, and no patient required ICU recovery. Two patients were readmitted within 30 days of discharge because of pneumothorax, and both were treated by chest-tube drain.

## 4. Discussion

A few studies have focused their attention on the role of nutritional status in patients with lung cancer and how preoperative immune-nutritional supports were useful in the improvement of postoperative outcomes by stimulating the immune system [17,18,19,20,21,22]. However, to date, no data was available concerning the impact of ONS on postoperative complications after uVATS lung resection.

The present study has three novel findings. First, it demonstrated that ONS assumption and arrythmia were independent predictors of complications in patients undergoing uVATS anatomical lung resection for cancer. Specifically, our results showed the benefit of preoperative ONS intake, in that it showed a positive impact on the postoperative outcomes, with a significant reduction of CPCs.

Second, our data showed that after WR, ONS intake did not improve postoperative outcomes; this was due to the minor surgical trauma and the plausible reduced immune reaction after surgery, as well as the very limited number of complicated cases that may underestimate its real clinical impact.

Finally, the third novel finding was the role of this specific ONS treatment (enriched with arginine, omega-3 fatty acids, and nucleotides) which appeared to be a promising immune–nutritional supplement for non-malnourished patients undergoing elective thoracic surgery, even if received for only 5–7 days before surgery.

As already reported in the literature [12], ONS has shown promising results, mainly in patients undergoing digestive surgery, demonstrating that the risks of postoperative complications and mortality after surgery were significantly reduced in patients who used ONS. Shoji et al. [23], in their prospective study, showed that ONS drastically improved immune–nutritional condition in 40 patients undergoing elective thoracic surgery. Indeed, they demonstrated that short-term preoperative ONS supplements (from 7 to 14 days before surgery) improved immune-nutritional parameters before surgery (i.e., blood lymphocyte count, serum albumin level, and body weight). However, no data regarding surgical details (type of resection, approach VATS vs. open) and postoperative outcomes were reported; moreover, only patients with poor immune-nutritional criteria were enrolled.

Kaya et al. [24], instead, demonstrated the positive effects of preoperative ONS with arginine, omega-3 fatty acids, and nucleotides in decreasing the complications and chest-tube removal time in 31 patients undergoing anatomic lung resection for NSCLC. In this prospective study, as well as in our study, patients who were malnourished (BMI < 18.5 kg/m^2^) or with metabolic disorders were excluded, but, unlike our study population, 20/31 patients (64.5%) were treated with an open approach.

The benefits of ONS after uVATS anatomic lung resection shown in our study were in line with those reported in the literature. However, concerning the impact of ONS after non-anatomic lung resection, we found that ONS intake did not improve postoperative outcomes. This can be explained both by the minor surgical trauma and by the consequent reduced immune reaction after surgery [12,23], as well as by the very limited number of complicated cases (twelve patients, of which only six received preoperative ONS), that did not allow for a full interpretation of the independent predictive impacts of the findings.

Moreover, in the present study, considering the good clinical condition of the enrolled patients (ASA ≤ 3); the improved nutritional status of this study population, compared with the population generally undergoing digestive surgery (we excluded malnourished patients with BMI < 18.5 kg/m^2^); and the very short-term action of ONS (5–7 days), we hypothesized that the ONS range of action was mostly impactful on the immunological condition rather than on the nutritional patient-status, that would have required a longer treatment.

In fact, it was assumed that the chronic production of inflammatory cytokines induced by cancer and surgery leads to metabolic alterations and the dysfunction of host homeostasis [25], increasing the patient’s susceptibility to postoperative complications [26]. In particular, it has been demonstrated that after surgery, IL-2 and IFN-γ (produced by Th1 cells) secretion levels were diminished, and those of IL-4 and IL-10 (produced by Th2 cells) were elevated. This shift in the Th1/Th2 balance toward a Th2-mediated immune response in the early postoperative stage may predict the occurrence of postoperative complications [6,8].

In this context, some authors have reported that due to the immunological ability of certain nutrients, mainly arginine, omega-3 fatty acids, and nucleotides, the ONS was able to modulate inflammation and to improve immune function, regulating the production of cytokines and limiting the immune reaction after surgery [21,24,27,28]. This implied a reduction of postoperative adverse events, specifically in terms of reductions in infections, morbidity, wound complications, and hospital stay [9,12].

Specifically, Iwase et al. showed that ONS enriched in omega-3 fatty acids impacted on the immunological conditions of patients undergoing cardiac surgery, even if used for 5 successive days before surgery [8]. Indeed, they demonstrated that ONS caused an increase of HLA-DR expression on monocytes, CD4/CD8 ratio, and IFN- γ production (a Th1 cytokine) and a decrease in the plasma levels of IL-10 (a Th2 cytokine). For all these reasons, guidelines [10,11] strongly recommend the assessment of nutritional status before and after major surgery for cancer and the encouragement of patients who do not meet their energy needs from normal food to take ONS (particularly those with arginine, omega-3 fatty acids, and nucleotides) for 5 to 7 days preoperatively, in order to avoid unnecessary hospitalization and to lower the risk of postoperative complications.

However, there is currently no clear evidence for the use of these ONS exclusively in the preoperative period or in non-malnourished patients undergoing major or minor VATS lung resection for cancer.

Indeed, the different ONS used in the studies, the different subgroups of surgical populations analyzed, and the different durations of ONS intake in the preoperative phase, together with the rarity of published studies focused on this topic, did not allow for a standardized immune–nutritional program able to define an optimal pathway of care for patients with lung cancer undergoing uVATS lung resection.

To the best of our knowledge, this is the first study focused on the association of ONS intake, in the context of an EPC program, with postoperative outcomes after uVATS lung resection, and the largest study on the use of ONS in thoracic surgery. However, our findings have limitations: this is a retrospective, single center, pathophysiology-driven clinical study. Before the present work, no study was available that formally defined the role of ONS within the EPC program in U-VATS patients.

The study time frame was short by design, and the number of observed events was limited, and hence it cannot fully elucidate the independent predictive impact of the findings without incurring a risk of model overfitting. Moreover, the applicable population for the ONS was selected, excluding patients with contraindications such as chronic renal failure and obesity.

Finally, considering the primary aim of the study and its retrospective nature, causal relationships could not be determined, and therefore, multicenter randomized studies are required to verify the clinical impact of ONS on postoperative outcomes in a broader population, as well as to optimize the ingredients, the intervention duration, and to improve the prognoses of patients undergoing elective uVATS lung resection for cancer.

Therefore, the results of this observational non-randomized study should be regarded as hypothesis-generating, deserving of further confirmatory studies.

## 5. Conclusions

In conclusion, this is the first study regarding the role of ONS as a predictor of postoperative complications after uVATS lung resection, and the largest study on the use of ONS in thoracic surgery.

Specifically, our results demonstrated that preoperative ONS use, in the context of an EPC program, is encouraging and promising in thoracic surgery, reducing the CPCs, compared with patients without nutritional supplementation.

Indeed, we found that ONS assumption was an independent predictor of complications after uVATS anatomic lung resection but, on the other hand, after WR, it did not influence postoperative outcomes.

Therefore, even if we reported only preliminary results on the predictive role of ONS in a selected population, we may propose, after adequate validation with large-scale randomized studies, that all patients scheduled for lung resection be carefully evaluated by a nutritionist before undergoing surgery.

## Figures and Tables

**Table 1:** Univariate analysis for predictors of postoperative cardiopulmonary complications after U-VATS anatomic lung resection.

Variable *	uVATS Anatomic Resection (277)		*p* Value
	CPC, Yes	CPC, No	
ONS	8 (2.9)	103 (37.2)	0.04
Sex, Male	13 (4.6)	122 (44.0)	0.25
Hypertension	19 (6.8)	149 (53.7)	0.70
Heart Ischemia	1 (0.3)	24 (8.6)	0.20
Arrhythmia	10 (3.6)	22 (7.9)	<0.01
CVD	4 (1.4)	15 (5.4)	0.20
CKD	4 (1.4)	23 (8.3)	0.62
CLD	3 (1.0)	14 (5.0)	0.45
DIA	8 (2.8)	34 (12.2)	0.12
Age, yo	70.4	67.7	0.11
BMI, kg/m^2^	28.4	26.4	0.03
FEV1%	98	97	0.77
DLCO%	74.2	76.1	0.52
ASA	1.4	1.3	0.44

* Numeric variables are expressed as ‘mean’; binary variables are expressed as ‘yes, *n* (%)’. CPC: cardiopulmonary complication, ONS: oral nutritional supplementation, CVD: cerebrovascular disease, CKD: chronic kidney disease, CLD: chronic liver disease, DIA: diabetes, BMI: body mass index, FEV1: forced expiratory volume in 1 s, DLCO: carbon monoxide diffusing capacity, ASA: American Society of Anesthesiologists.

**Table 2:** Stepwise logistic regression for predictors of postoperative cardiopulmonary complications after U-VATS anatomic lung resection.

Cardiopulmonary Complications	Coef.	Std. Err.	*p* Value	[95% Conf. Interval]	
Age, y.o.	0.0036095	0.0020991	0.09	−0.0005231	0.007742
Arrhythmia	0.2042478	0.0595694	0.001	0.0869741	0.3215216
ONS	−0.0753747	0.0391459	0.05	−0.1524409	0.0016915

ONS: oral nutritional supplementation.

**Table 3:** Univariate analysis for predictors of postoperative cardiopulmonary complications after U-VATS wedge resection.

Variable *	uVATS Wedge Resection (214)		*p* Value
	CPC, Yes	CPC, No	
ONS	6 (2.8)	87 (40.6)	0.64
Sex, Male	6 (2.8)	96 (44.8)	0.86
Hypertension	11 (5.1)	114 (53.2)	0.02
Heart Ischemia	3 (1.4)	20 (9.3)	0.10
Arrhythmia	2 (0.9)	31 (14.4)	0.90
CVD	1 (0.4)	15 (7.0)	0.90
CKD	3 (1.4)	27 (12.6)	0.26
CLD	0 (0)	22 (10.2)	0.23
DIA	3 (1.4)	31 (14.4)	0.37
Age, yo	72.6	66.6	0.09
BMI, kg/m^2^	27.8	25.9	0.15
FEV1%	79.7	95	0.01
DLCO%	70.8	74.8	0.42
ASA	1.5	1.3	0.51

* Numeric variables are expressed as ‘mean’; binary variables are expressed as ‘yes, *n* (%)’. CPC: cardiopulmonary complication, ONS: oral nutritional supplementation, CVD: cerebrovascular disease, CKD: chronic kidney disease, CLD: chronic liver disease, DIA: diabetes, BMI: body mass index, FEV1: forced expiratory volume in 1 s, DLCO: carbon monoxide diffusing capacity, ASA: American Society of Anesthesiologists.

**Table 4:** Baseline characteristics of the enrolled patients.

Variable *	ONS (204)	Not-ONS (287)	All Patients (491)	*p* Value
AGE, yo	69.0 (10.1)	67.6 (10.4)	67.6 (10.3)	0.01
SEX, Male	103 (50.5)	134 (46.7)	237 (48.2)	0.40
BMI	24.2 (3.9)	27.9 (4.7)	26.4 (4.7)	<0.01
FEV1%	97.2 (20.1)	94.8 (20.1)	95.8 (20.1)	0.19
DLCO%	73.1 (15.8)	76.9 (17.2)	75.3 (16.7)	0.01
ASA	1.4 (0.7)	1.3 (0.6)	1.3 (0.6)	0.47
Hypertension	125 (61.3)	168 (58.5)	293 (59.6)	0.54
Heart Ischemia	19 (9.3)	29 (10.1)	48 (9.7)	0.77
Arrhythmia	23 (11.3)	42 (14.6)	65 (13.2)	0.27
CVD	18 (8.8)	17 (5.9)	35 (7.1)	0.21
CKD	19 (9.3)	38 (13.2)	57 (11.6)	0.18
CLD	14 (6.9)	25 (8.7)	39 (7.9)	0.45
DIA	36 (17.6)	40 (13.9)	76 (15.4)	0.26
Type resection				0.78
Lobectomy	91 (44.6)	141 (49.1)	232 (47.2)	
Segmentectomy	18 (8.8)	23 (8.0)	41 (8.3)	
Bilobectomy	2 (1)	2 (0.7)	4 (0.8)	
Wedge resection	93 (45.6)	121 (42.2)	214 (43.5)	

* Numeric variables are expressed as ‘mean (SD)’; binary variables are expressed as ‘yes, *n* (%)’. SD: standard deviation, ONS: oral nutritional supplementation, BMI: body mass index, FEV1: forced expiratory volume in 1 s, DLCO: carbon monoxide diffusing capacity, ASA: American Society of Anesthesiologists, CVD: cerebrovascular disease, CKD: chronic kidney disease, CLD: chronic liver disease, DIA: diabetes.

## Data Availability

The data underlying this article will be shared, on reasonable request to the corresponding author.
